# Psychosocial work environment and mental health among the global workforce of seafarers in the wake of the COVID-19 pandemic

**DOI:** 10.1186/s12889-023-17035-2

**Published:** 2023-11-03

**Authors:** Rebecca Hayes-Mejia, Martin Stafström

**Affiliations:** 1 Division of Social Medicine and Global Health, Lund University, Jan Waldenströms gata 35, Malmö, 21428 Sweden; 2 Marine Benefits AS, Bergen, Norway

**Keywords:** Seafarers, COVID-19, Mental health, Stress, Anxiety, Depression, International labor policy

## Abstract

**Background:**

The aim of this study was to investigate the impact of the COVID-19 pandemic on the mental health outcomes of international seafarers, who played a crucial role in maintaining global trade during the pandemic. The study examined how changes in psychosocial work environment and policies affected mental health outcomes among seafarers.

**Methods:**

We analyzed a survey including answers from 17,861 seafarers, serving on 44 different international commercial vessels with 154 different nationalities. Stress, anxiety, and depression were applied as outcome measures in this study. Three sets of independent variables were included; work-related consequences of the COVID-19 pandemic, general psychosocial work environment onboard, and socioeconomic variables. First, we applied binary linear regression, followed by a multivariate linear regression analysis.

**Results:**

The study found that changes in safety consciousness and clear communication from employers were associated with better mental health outcomes among seafarers. Eroded policies related to crew changes had a significant negative effect on mental wellbeing due to delays caused by national quarantine guidelines and travel restrictions. The results also showed a discrepancy in mental health outcomes between those onboard and those onshore, with stress being present in both groups.

**Conclusions:**

The findings suggest that crisis management within shipping companies played an important role in mitigating adverse mental health outcomes during the pandemic. Clear communication from employers and emphasizing safety issues onboard were effective strategies for promoting better mental wellbeing among seafarers. However, delays in crew changes had a significant negative impact on mental health outcomes, highlighting the need for global cooperation and overarching agreements to protect international seafarers during times of crises.

**Supplementary Information:**

The online version contains supplementary material available at 10.1186/s12889-023-17035-2.

## Introduction

The global supply chain allowing for the movement of international goods from one part of the world to the other is manned by seafarers. During the COVID-19 pandemic, when airports and land borders were closed or shut down, seafarers and their ships safeguarded the international trading routes, securing the global supply of necessary goods to sustain countries under lockdowns and restrictions. Unfortunately, the restrictions also applied to seafarers who were then stuck onboard ships for months on end, unable to return to their families and homes. As four UN agencies concluded in a joint statement: “This […] crisis has resulted in significant mental strain […] imperiling working conditions in the shipping sector” [1]. In addition, the most recent Review of Maritime Transport, the United Nations Conference on Trade and Development (UNCTAD) refers to the pandemic as “an unprecedented humanitarian crisis for seafarers” [2].

Overall, knowledge about the mental health and wellbeing of seafarers is limited. Seafarers have not been researched systematically despite their important role in the global supply chain. A small number of primary data studies have been published in the past decade. Oldenburg et al. [3] found moderate burnout risks among seafarers among those 251 that were surveyed. Song et al. [4] surveyed 668 Chinese seafarers, finding high levels of stress, particularly among them with fixed contracts. Slišković and Penezić [5] found that sleep deprivation and stress were prevalent among 530 surveyed Croatian seafarers. A mixed methods study including a survey with 1,504 respondents by Sampson and Ellis [6] found that seafarers’ mental health and wellbeing were sub-optimal. In a recent study by Pauksztat et al. [7], 504 seafarers were followed-up in terms of mental wellbeing in relation to the COVID-19 pandemic. The study found higher prevalence of anxiety and depression during the pandemic compared to before it, and could link these outcomes to longer shifts, longer stays onboard and to ships that carried flags of convenience.

Around 1.9 million seafarers operate over 74,000 vessels in the international trade. Among these about 850,000 are officers and 1,050,000 are ratings. It is estimated that just about 1% of the workforce are female seafarers. The Philippines, Russia, Indonesia, China, and India together supplies 44% of all seafarers globally [8]. The seafaring occupation comes with certain job demands that are singlehandedly challenging, and combined they may have cumulative or even synergistic negative effects on mental health outcomes [7]. For those experiencing negative outcomes from their psychosocial work environment, stress is a first sign, whereas anxiety and depression are more severe conditions [9]. Because seafarers work in shifts, they are exposed to the risks of a wide range of biological and psychological negative outcomes [10]. Additionally, because they socially isolated over an extended period of time, together with being exposed to a noisy and sometimes dangerous environment in extreme weather conditions, posing further risks to their mental wellbeing [3, 11]. In addition, their access to preventive and maintenance health care is limited [12].

One of the main consequences of the pandemic travel restrictions within the shipping industry was that it became more or less impossible to abide with the international treaties on crew scheduling [13]. Under normal circumstances, officers usually have contracts where they spend half the time onboard and the other half at home. Ratings, together with cooks and other non-officers, are hired on less secure contracts, usually serving six to eight months at sea and then renew their contracts after an extended leave onshore, normally three to four months. During the pandemic, these contract lengths were in many cases involuntarily extended, with 12-month onboard stays becoming a usual occurrence. In addition, quarantine rules restricted seafarers’ ability to go ashore while in port, further isolating them from life outside their vessel [14].

One additional impact of the abovementioned challenges were the financial difficulties that seafarers faced because they were unable to renew contracts. Seafarers are usually paid for the time they are onboard, with the exception of some officers. The travel and border restrictions put in place by national governments made it impossible for many seafarers to report for duty and thus to gain a living. This put families in low- and middle-income country settings under great financial stress.

It is imperative to understand what impact the pandemic had on seafarers and their wellbeing during the COVID-19 pandemic to secure international trade in future crises. The aim of this study is, thus, to investigate how different factors were associated with outcomes of stress, anxiety and depression among international seafarers during the COVID-19 pandemic. The main hypothesis being that the mental health among seafarers was affected by onboard COVID-19 mitigations strategies, independent from the general psychosocial environment.

## Methods

### Population and data sampling

The data collection was a joint venture between Lund University and Marine Benefits, a Norwegian insurance company providing health insurance for crews in international shipping. We invited a large number of shipping companies to participate in the study. In total, 44 different shipping companies chose to participate. The study was granted approval by the Swedish Ethical Review Authority. The research team forwarded a link to the online survey to each of the participating shipping companies. The shipping companies then relayed it by email to all their contracted seafarers, about 160,000. The survey link was accompanied by a formal invitation to the online survey, as well as information about the study purpose and their rights as respondents. The survey link was sent out four times in total to all participants, no matter if they had responded or not. In order to complete the survey, the seafarers had to give informed consent.

The questionnaire consisted of 41 questions. The first part was background questions (age, gender civil status etc.). Then followed a number of different psychometric scales (see below – measurements). The subsequent questions then measured different aspects of the psychosocial work environment, both in general and during the Covid-19 pandemic in particular. The questionnaire was only distributed in an English language version, as international seafarers are required to have a working knowledge in that particular language [15].

The data was collected in February to March 2022. In total, 28,105 seafarers gave informed consent to participate. 24,662 answered the first question. However, only 17,861 participants completed the full survey from start to finish, with seafarers dropping out of the survey at different points (see Table 1 for a flow chart of the data collection). For the subsequent analyses, we have included the seafarers that answered the questions included in each analysis respectively. This has the implication that some analyses include a larger sample size than others, particular the in presentation of the descriptive data (Tables 2 and 3), whereas the multivariate analyses only include those responding to the full survey.

**Table 1:** Continuous response rate across the survey

	n	Response rate
Informed consent	28,105	-
Question 1	24,662	87.75%
Question 11	23,951	85.22%
Question 21	22,237	79.12%
Question 31	18,468	65.71%
Question 41 (last question)	17,861	63.55%

**Table 2:** Descriptives of the continuous variables applied in the analyses

	Total					At sea					Onshore					t-test*	Wilcoxon*
	n	Mean	SD	% indicated		n	Mean	SD	% indicated		n	Mean	SD	% indicated		p-value	p-value
Stress	20,830	15.87	4.66			11,004	16.06	4.65			9826	15.65	4.65			p < 0.001	
Anxiety	20,087	3.09	3.94	5.60		10,630	3.21	4.06	6.18		9457	2.95	3.79	4.94		p < 0.001	0.0001
Depression	19,430	3.44	4.40	8.34		10,298	3.69	4.51	9.13		9132	3.16	4.25	7.45		p < 0.001	p < 0.001
Time onboard/onshore (months)	23,951	3.03	1.96			12,634	2.79	1.63			11,317	3.29	2.27			p < 0.001	
Employer COVID-19 measures scale	17,861	31.39	5.69			9426	31.02	5.81			8435	31.81	5.52			p < 0.001	

*Tests of statistical difference between those at sea and those onshore, for Anxiety (GAD-7) and Depression (PHQ-9) we also tested the proportion of those being indicated as being anxious and depressed between those at sea and those onshore applying the Wilcoxon rank test

**Table 3:** Descriptives of the main exposure variables

Variable	Category	Total			At sea			Onshore		χ2*
		n	%		n	%		n	%	
Experienced crew change	No	7285	39.60		4000	41.04		3285	37.97	< 0.001
delays past 6 months	Yes, while at home	4537	24.66		2491	25.56		2046	23.65	
	Yes, while onboard	6576	35.74		3256	33.41		3320	38.38	
Due to COVID-19:	Strongly disagree	1275	7.07		730	7.66		545	6.40	< 0.001
There been a positive	Disagree (a bit worse)	1940	10.75		985	10.34		955	11.22	
change in routines?	No (the same as pre-covid)	3978	22.05		2138	22.43		1840	21.62	
	Agree	8749	48.49		4666	48.96		4083	47.97	
	Strongly agree	2100	11.64		1011	10.61		1089	12.79	
Due to COVID-19:	Strongly disagree	282	1.56		163	1.71		119	1.40	< 0.001
I have become more	Disagree (a bit worse)	297	1.65		156	1.64		141	1.66	
safety conscious	No (the same as pre-covid)	2334	12.94		1263	13.25		1071	12.58	
	Agree	897	49.72		4918	51.61		4052	47.60	
	Strongly agree	6159	34.14		303	31.79		3129	36.76	
Due to COVID-19:	Strongly disagree	431	2.39		236	2.48		195	2.29	0.019
My workload has	Disagree (a bit worse)	678	3.76		351	3.68		327	3.84	
increased significantly	No (the same as pre-covid)	6861	38.03		3728	39.12		3133	36.81	
	Agree	7921	43.90		4109	43.12		3812	44.78	
	Strongly agree	2151	11.92		1106	11.61		1045	12.28	
Due to COVID-19:	Strongly disagree	888	4.92		518	5.44		370	4.35	0.003
Now I have more social	Disagree (a bit worse)	1546	8.57		804	8.44		742	8.72	
interaction onboard	No (the same as pre-covid)	6777	37.56		3567	37.43		3210	37.71	
	Agree	7562	41.91		4008	42.06		3554	41.75	
	Strongly agree	1269	7.03		633	6.64		636	7.47	
Due to COVID-19:	Strongly disagree	1059	5.87		611	6.41		448	5.26	< 0.001
The social atmosphere	Disagree (a bit worse)	2304	12.77		1183	12.41		1121	13.17	
has improved	No (the same as pre-covid)	5098	28.26		2746	28.81		2352	27.63	
	Agree	8172	45.29		4301	45.13		3871	45.48	
	Strongly agree	1409	7.81		689	7.23		720	8.46	

*The *χ2*-test tested the statistical difference between those at sea and those onshore

Overall, access to the Internet varies across different fleets. The larger shipping companies offer free and unlimited access to satellite supported Internet onboard. However, at very low speeds and oftentimes the connection is weak, making an online survey like ours difficult to fill out. This could partly explain the rather large dropout rate through the survey.

### Measurements

In this study we have applied three different outcome measures based on validated psychometric scales. One measuring stress, anxiety, and depression respectively.

Stress was measured using the Perceived Stress Scale (PSS-10) [16–18]. The scale includes ten items ranging from zero to four points each, giving a range from 0 to 40 in the full scale with a normal distribution (mean = 15.9, SD = 4.7, see Table 2). The scale showed a sufficient internal reliability with a Cronbach’s alpha of 0.720. The higher the score on PSS-10, the higher the level of stress. When making comparisons with other populations, it is recommended that the mean score is completed and evaluated [18, 19].

Anxiety was measured using the Generalized Anxiety Disorder scale (GAD-7) [20, 21]. The scale includes seven items ranging from zero to three points each, giving a range from 0 to 21 in the full scale. As the distribution was log-normal (mean = 3.1, SD = 3.9, see Table 2), we log-transformed the scale in order to apply linear models in the analyses. The scale showed an excellent internal reliability with a Cronbach’s alpha of 0.916. The diagnostic criteria for the GAD-7 scale is eight or higher [22, 23].

Depression was measured using the Patient Health Questionnaire (PHQ-9) [24]. The scale includes nine items ranging from zero to three points each, giving a range from 0 to 27 in the full scale. As the distribution was log-normal (mean = 3.4, SD = 4.4, see Table 2), we log-transformed the scale in order to apply linear models in the analyses. The scale showed a very good internal reliability with a Cronbach’s alpha of 0.887. The scale diagnoses major depression if five or more of the nine symptom criteria in the scale have been present at least “more than half the days” in the past two weeks, and at least one of the symptoms is depressed mood or anhedonia is present [24].

We included three sets of independent variables. The first set of variable addresses work-related consequences of the COVID-19 pandemic, representing the main exposure variables in the analyses. The second set assesses the general psychosocial work environment onboard, and the third were socioeconomic variables.

The main exposures in relation to our aim were a set of variables directly linked to the COVID-19 pandemic. The first variable indicates if they had experienced delays in crew changes over the past 6 months. Then the respondents had to indicate in the questionnaire to what extent they agreed or disagreed on five different statements about how the situation had changed onboard during the pandemic – see Appendix 1. Furthermore, we analyzed a scale that we constructed ourselves based on eight different statements (see Appendix 1 for additional documentation) about how their employer had managed pandemic related challenges. The respondents had to indicate to what degree they agreed with the statements on a Likert-scale ranging from strongly disagree to strongly agree. The scale proved to have a very high internal validity (Cronbach’s alpha 0.909), and a confirmatory factor analysis suggested to include all variables into the scale.

In order to assess the general psychosocial work environment onboard we included eight variables – see Appendix 1 for questions. We also included the Employee Satisfaction Index [24]. The scale assesses the overall professional engagement among employees, and categorizes the respondents into five different groups: Subversive, Dysfunctional, Ambivalent, Engaged, Highly Engaged.

Age, rank and nationality were the variables that made up the socioeconomic set of variables. *Age* was categorized into 10-year intervals, except the youngest category that included 13 years, ranging from age 18–30. *Rank* was divided into 12 different categories, based on the seafarers’ self-reported onboard placement for the most recent contract agreement. *National origin* was indicated in free text by the respondents. We then coded these different nationalities – with the exception of the four most common nationalities of Filipino, Indians, Russians and Ukrainians – into ten different continental/regional categories: EU, non-EU Europe, North Africa, Sub-Saharan Africa, The Middle-East/Gulf States and Central Asia, Sub-Continental Asia, East Asia, Oceania, North America, Latin America and, finally, the Caribbean. We then included a variable of where the seafarers currently were based – at sea or onboard – and another indicating how long time they had spent in their current situation, which was included as an independent variable in the initial multivariate regression analyses (Table 4), and then as a stratifier in the following (Table 5).

**Table 4:** Multivariate linear regression analyses of the association between stress, anxiety, and depression and the main exposure variables, adjusting for the socioeconomic background variables (Model 1) and both socioeconomic background variables and psychosocial work environment conditions (Model 2), applying 95% confidence intervals

	Stress						Anxiety						Depression				
	Model 1			Model 2			Model 1			Model 2			Model 1			Model 2	
	Coeff.	p		Coeff.	p		Coeff.	p		Coeff.	p		Coeff.	p		Coeff.	p
Experienced crew change delays past 6 months	0.213	< 0.001		0.123	< 0.001		0.047	< 0.001		0.035	< 0.001		0.047	< 0.001		0.028	< 0.001
There has been a positive change in routines	− 0.191	< 0.001		− 0.086	0.135		− 0.026	0.004		− 0.013	0.106		− 0.019	0.033		− 0.005	0.497
I have become more safety conscious	− 0.495	< 0.001		− 0.277	< 0.001		− 0.076	< 0.001		− 0.041	< 0.001		− 0.087	< 0.001		− 0.047	< 0.001
My workload has increased significantly	0.785	< 0.001		0.392	< 0.001		0.111	< 0.001		0.045	< 0.001		0.116	< 0.001		0.037	< 0.001
Now I have more social interaction onboard	− 0.038	0.419		0.088	0.005		− 0.048	< 0.001		− 0.036	< 0.001		− 0.020	0.052		− 0.006	0.519
The social atmosphere has improved	0.208	< 0.001		0.177	< 0.001		0.016	0.131		0.011	0.238		− 0.005	0.626		− 0.009	0.325
Employer COVID-19 measures scale	− 0.137	< 0.001		− 0.068	< 0.001		− 0.018	< 0.001		− 0.008	< 0.001		− 0.025	< 0.001		− 0.013	< 0.001
Being at sea	0.355	< 0.001		0.283	< 0.001		0.038	0.013		0.024	0.101		0.088	< 0.001		0.087	< 0.001
*Model R-squared*	*0.101*			*0.234*			*0.071*			*0.215*			*0.096*			*0.274*	

**Table 5:** Stratified multivariate linear regression analyses of the association between stress, anxiety, and depression and the main exposure variables for those at sea and those onshore respectively, adjusting for both socioeconomic background variables and psychosocial work environment conditions (Model 2), applying 95% confidence intervals

	**Stress**				Anxiety				**Depression**			
	At sea		Onshore		At sea		Onshore		At sea		Onshore	
	Coeff.	p	Coeff.	p	Coeff.	p	Coeff.	p	Coeff.	p	Coeff.	p
Experienced crew change delays past 6 months	0.148	0.004	0.104	0.05	0.045	< 0.001	0.0144	0.246	0.044	< 0.001	0.009	0.455
There has been a positive change in routines	-0.116	0.021	0.022	0.679	-0.015	0.167	0.004	0.743	-0.011	0.286	0.001	0.503
I have become more safety conscious	-0.222	< 0.001	-0.193	0.006	-0.025	0.071	-0.031	0.052	-0.038	0.004	-0.033	0.032
My workload has increased significantly	0.478	0.201	0.246	< 0.001	0.053	< 0.001	0.023	0.096	0.051	< 0.001	0.011	0.407
Now I have more social interaction onboard	0.078	< 0.001	0.174	0.006	-0.046	0.001	-0.004	0.796	-0.005	0.709	0.009	0.512
The social atmosphere has improved	0.222	< 0.001	0.149	0.019	0.016	0.213	0.015	0.307	-0.014	0.237	0.002	0.874
Employer COVID-19 measures scale	-0.025	0.009	-0.041	< 0.001	-0.005	0.027	-0.001	0.65	-0.012	< 0.001	-0.003	0.211
*Model R-squared*	*0.264*		*0.244*		*0.253*		*0.204*		*0.309*		*0.261*	

### Statistical analysis

All estimates and confidence intervals (CI) were computed using STATA 16. Initially, we performed descriptive data analysis, which included testing the difference between those at sea and those onshore either with t-test or chi-square test. We then conducted multivariate linear regression analyses for each outcome variable respectively, performed in two steps. The first step only included the main exposure variables and the socioeconomic background indicators. In the next and final step, we included the general psychosocial work environment indicators. This approach was applied to test for possible confounding between general and pandemic-related circumstances. In a final analysis, we stratified the full linear regression models for those being at sea and those onshore in order to better assess how posting station affected the outcomes. For analytical purposes, national origin, and rank were added to the regression models as categorical variables, whereas the Likert-scale variables were added as continuous ones.

## Results

The results (Table 2) indicated that the seafarers currently at sea rated their mental health differently from the ones onshore. Overall, the outcome variables were all more present among those at sea, and for the main exposure variables the onshore seafarers had a more positive recollection of how the COVID-19 pandemic had affected working conditions.

The multivariate regression analyses indicated that all of the main exposure variables were independently associated with stress (Table 4). It was also noteworthy that changes in social interaction were not significantly associated with stress in the initial model, while it was in the full model. This indicates that this association was mediated by the general psychosocial conditions onboard.

For anxiety (Table 4), the regression analyses suggested that all of the main exposure variables were independently associated with a higher degree of anxiety, with the exception of improved routines and social atmosphere. There were signs of confounding, particularly for the employer COVID-19 scale, between the main exposure variables and the general psychosocial working conditions.

The multivariate regression analyses with depression as the dependent variable (Table 4) indicated that experience of delay in crew changes, safety consciousness, workload and the employer COVID-19 measure were independently associated with depression. It was also noteworthy that improved routines, social interaction and atmosphere were not significantly associated with depression in the final model, while both improvements in routines and social interaction were in the initial model. This indicates that these association were mediated by the general psychosocial conditions onboard.

The location of the seafarer was associated with stress and depression, but not anxiety. This led us to further investigate the importance of location through stratifying the analyses. When stratifying the multivariate regression analyses based on the location of the seafarers, the findings indicated that there were more similarities in regards to stress than for anxiety and depression (Table 5). It also became apparent, that there were indeed different risk patterns for anxiety between those at sea and those onshore.

## Discussion

The findings of the present study clearly indicate that the psychosocial work environment within the maritime industry is associated with mental health outcomes, such as stress, anxiety, and depression. In addition, the analyses suggest that the work environment measures taken to adapt to the COVID-19 pandemic had an independent association with these mental health outcomes.

When putting the outcome measures in our sample in relation to other populations, it shows that international seafarers are more stressed (PSS-10) than a general population sample in Sweden and in the US [18, 25]. For both anxiety (GAD-7) and depression (PHQ-9) we see a similar pattern, where the prevalence of anxiety among the seafarers are similar to the general population samples – e.g., in Sweden and Australia – or somewhat higher – e.g., in Germany and Belgium [16, 26–28], but somewhat lower than in primary health care settings among health care seekers in the US [23, 24]. It should be noted that the concept of mental health varies across cultures and it is, therefore, difficult to make inconclusive comparisons between one particular setting and a global population [29].

There are a number of studies that have studied the mental health of international seafarers, particularly during the Covid-19 pandemic [30–32]. These studies find similar levels, or slightly higher levels of stress, anxiety, and depression compared to our results. However, they did not apply the same psychometric measurements, nor did they include larger samples of seafarers.

The finding that the employers’ ability to communicate effectively, clearly, and transparently with seafarers was associated with better mental health outcomes. This indicates that the employers had agency to influence the mental health onboard by how they implemented their mitigation strategies put in place during the pandemic. Similar findings have been found in several studies among other frontline workers, where it has been suggested that the association is either found because good organizational support and communication buffered negative consequences of mitigation strategies [33], but also that it affected the overall approach – either an optimistic or pessimistic one – to the new challenges [32, 34].

Our finding that changes in safety consciousness during the pandemic was associated with mental health outcomes could also be linked to the above relationship. In addition, previous studies have shown that there is a direct link between crew retention and safety at sea [35]. Hence, this suggests that low safety consciousness leads to a poor psychosocial environment. This implies that, in a business where safety consciousness has become engrained in the culture [36], emphasizing safety could be interpreted as a way to exacerbate company culture and professional pride [37]. Continuing this line of thought, focusing on safety would then have the same effect as a buffer of negative psychosocial work environment as structured communication and support in general [33].

Research points at the social environment being a vital determinant of mental health [38], particularly professional settings [39]. Yet, in our data the policy indicators were in general associated with all three mental health outcomes, whereas this was not the case for social interaction and atmosphere. We assume that this pertains to the need, especially when being on a ship for months, to have clear structures and routines to attach to one’s daily routines, which seems to buffer adverse impact of stress on mental health outcomes [40].

Overall, there was a clear discrepancy in the results, particularly in relation to anxiety and depression, between those onboard and those onshore. This is by no means surprising, as mental health outcomes vary over time and are negatively associated with being on active duty or on vacation [41]. That the associations with stress were still quite present could very well be an indicator of vacation related stress [42, 43], but also due to additional financial stress.

There are several policy implications of this study. First, the results indicate that the shipping companies’ mitigation strategies, particularly having clear and transparent communication with the onboard crews, had a positive impact on mental wellbeing. The other components of the mitigation strategies are to foster and facilitate a positive working environment, focus on accident awareness and prevention, provide adequate medical care and health services whenever necessary, either onboard or at home, as well as keeping seafarers’ families updated and showing them concern. However, the most important policy issue to resolve would be to enable crew changes, also during times of crisis [44]. The results from our study clearly indicates that the delays that happened during the pandemic had a significant negative effect on mental wellbeing, as it both affected those onboard – but also had an immense impact on those onshore – affecting the livelihood of a great number of people especially in low- and middle-income country settings. In conclusion, based on the findings from this study, and in line with previous research, we recommend shipping companies to develop a health promoting workplace for seafarers with a focus on preventative measures, as this is shown to have larger impact on the wellbeing of crew [31, 45, 46].

### Strengths and limitations

The most obvious strength with this study is found in its sheer numbers. No other survey in the past has survey this many seafarers at the same time with the same instrument. In addition, the survey included seafarers from 154 different nationalities and with some exceptions – mainly the participation rate among Indonesian and Chinese seafarers – the proportion of these nationalities was representative of the workforce at large. The same can be said of the proportion of officers and ratings as well as age and gender. Yet, there were some limitations to the sampling. First, only 44 shipping companies volunteered to participate, even though these included some of the very largest companies in the world, many companies, especially mid and small-sized ones were missing. In addition, some vessel types were less represented than others – mainly cruise ships, which can be explained by the interruption in cruise ship operations during the pandemic.

Another strength with this study was that it applied validated scales as measurements of the outcome variables. A common challenge in mental health epidemiology is the vast range of different psychometric scales available to researchers. In this case, we decided to choose well-established validated measurements that could identify levels of stress, anxiety and depression in a general population sample. It should be noted that we at no point applied these scales in order to diagnose or triage the respondents.

The major limitation of this study was the low response rate. Of the invited 160,000 seafarers, a little more than 10% completed the full survey. We could not perform any analysis of those who chose not to participate in the survey. However, there were no statistically significant differences in terms of age, sex, and current location among those who did not complete the survey and those who did. On the other hand, those who actually completed the survey had a statistically significant higher probability to be officers and having spent shorter time at their current posting. Even more importantly, for the scales measuring stress, anxiety, and depression the mean scores were statistically significantly higher among those not completing the survey, indicating that the findings underestimate the associations between psychosocial work environment and mental health outcomes.

Another apparent limitation to this study was the cross-sectional design that does not allow us to draw any conclusions about inference between the psychosocial work environment and mental health outcomes.

As we wanted to keep the questionnaire as short as possible, expecting the decline in response rate throughout the survey, we opted to exclude a more comprehensive scale on psychosocial work environment. This has a few repercussions, mainly that the operationalization of the psychosocial work environment is based on symptoms rather than perceptions. This suggest that we might have assessed the psychosocial work environment to be better than it actually is, also leading to lower estimates and potentially suboptimal regression modelling.

## Conclusion

This study found an association between different changes to the work environment – particularly those pertaining to onboard policies – and mental health outcomes among international seafarers during the COVID-19 pandemic. The results suggest that these factors were significantly independent, yet confounded by the general psychosocial environment.

The study results suggest that crisis management within shipping companies were an important factor to mediate adverse mental health outcomes during the COVID-19 pandemic. The inability to globally address the difficulties in carrying out crew changes due to national quarantine guidelines and travel restrictions had a negative effect on mental health outcomes. As a global workforce international seafarers need to be protected by overarching global agreements in times of crises.

### Electronic supplementary material

Below is the link to the electronic supplementary material.

Supplementary Material 1

## Data Availability

The dataset used and analyzed during the current study is available from the corresponding author on reasonable request. The Individual participant data that underlie the results reported in this article, together with study protocols and analytical coding will be shared if a number of requirements are met. First, we require a methodologically sound proposal from the researcher in question. In addition, we require that data storage will be secure, and, finally, that the purpose is for scientific reasons only. Data will be made available 3 months after the publication of this article up to 36 months after.

## Abbreviations

EU: The European Union
GAD: 7–Generalized Anxiety Disorder Scale
PHQ: 9–Patient Health Questionnaire
PSS: 10–Perceived Stress Scale
UN: United Nations
UNCTAD: United Nations Conference on Trade and Development
