# Supplementary material for: Psychosocial work environment and mental health among the global workforce of seafarers in the wake of the COVID-19 pandemic
Source: BMC Public Health. 2023 Nov 3;23:2151. doi: 10.1186/s12889-023-17035-2 (PMC10623868; doi:10.1186/s12889-023-17035-2)
Supplement: Supplementary file 1 — Supplementary Material 1 [file 12889_2023_17035_MOESM1_ESM.docx]

**Appendix 1**

**Questions on changing work environment due to the COVID-19 pandemic**

1. Have the routines improved onboard because of COVID-19?
2. Have you yourself become more conscious about safety onboard because of COVID-19?
3. Has the workload onboard become worse because of COVID-19?
4. Has the social atmosphere onboard improved because of COVID-19?
5. Has the social interaction onboard improved because of COVID-19?

The Likert-scale answer alternatives to the above questions were:

I strongly disagree - I disagree - No, it has not changed - I agree - I strongly agree.

**The employer pandemic management scale**

The following statements were included based on the initial statement:

During the COVID-19 pandemic, how have you experienced your employer’s actions when it comes to the following?

1. My employer has been transparent and provided me with regular and updated information throughout the pandemic.
2. My employer has done its best to carry out crew changes in a safe and timely manner.
3. I believe my employer supports lobby efforts to promote the recognition of seafarers as key workers.
4. My employer has provided adequate medical care and health services whenever necessary during the pandemic, both onboard and at home.
5. My employer had provided us with adequate Wi-Fi connection and internet access.
6. My employer has fostered and facilitated a more positive working environment during the covid-19 pandemic (e.g., team building, anti-bullying, stress reduction, coaching, social gatherings, etc.)
7. My employer had increased its focus on accident awareness and prevention.
8. My employer keeps my family updated and shows sincere care for their wellbeing.

The Likert scale answer alternatives were:

Strongly disagree – Disagree – Not sure – Agree – Strongly agree

We performed a confirmatory factor analysis, which generated one factor with an Eigen value greater than 1. All variables loaded into this factor with loads ranging from 0.593 to 0.819, indicating that all variables were contributing to the interpretation of the factor.

**General psychosocial work environment**

The respondents were asked to rate to what extent they agreed to the following statements:

1. I feel lonely
2. I have at least one co-worker to talk to
3. I feel bullied
4. I feel discriminated against
5. We have a lot of group activities while onboard
6. I get enough sleep
7. I get enough rest throughout the day.

The answer alternatives for these claims were: At no time, Some of the time, Less than half of the time, More than half of the time, Most of the time, All of the time.
